# Impaired verbal memory in Parkinson disease: relationship to prefrontal dysfunction and somatosensory discrimination

**DOI:** 10.1186/1744-9081-5-49

**Published:** 2009-12-15

**Authors:** Stephan Bohlhalter, Eugenio Abela, Dorothea Weniger, Bruno Weder

**Affiliations:** 1 Department of Neurology, Division of Cognitive and Restorative Neurology, University Hospital Bern, Bern, Switzerland; 2 Department of Neurology, Kantonsspital St. Gallen, St.Gallen, Switzerland; 3 Department of Neurology, University Hospital Zurich, Zürich, Switzerland; 4 Department of Neurology, University of Bern, Bern, Switzerland

## Abstract

**Objective:**

To study the neurocognitive profile and its relationship to prefrontal dysfunction in non-demented Parkinson's disease (PD) with deficient haptic perception.

**Methods:**

Twelve right-handed patients with PD and 12 healthy control subjects underwent thorough neuropsychological testing including Rey complex figure, Rey auditory verbal and figural learning test, figural and verbal fluency, and Stroop test. Test scores reflecting significant differences between patients and healthy subjects were correlated with the individual expression coefficients of one principal component, obtained in a principal component analysis of an oxygen-15-labeled water PET study exploring somatosensory discrimination that differentiated between the two groups and involved prefrontal cortices.

**Results:**

We found significantly decreased total scores for the verbal learning trials and verbal delayed free recall in PD patients compared with normal volunteers. Further analysis of these parameters using Spearman's ranking correlation showed a significantly negative correlation of deficient verbal recall with expression coefficients of the principal component whose image showed a subcortical-cortical network, including right dorsolateral-prefrontal cortex, in PD patients.

**Conclusion:**

PD patients with disrupted right dorsolateral prefrontal cortex function and associated diminished somatosensory discrimination are impaired also in verbal memory functions. A negative correlation between delayed verbal free recall and PET activation in a network including the prefrontal cortices suggests that verbal cues and accordingly declarative memory processes may be operative in PD during activities that demand sustained attention such as somatosensory discrimination. Verbal cues may be compensatory in nature and help to non-specifically enhance focused attention in the presence of a functionally disrupted prefrontal cortex.

## Background

Non-demented Parkinson's disease (PD) has been associated with a number of neurocognitive deficits including executive and memory dysfunction [1,2]. Executive functions, generally defined as the ability to plan, monitor, and carry out goal directed behaviour in response to changing environmental situations, rely on intact frontal lobe processes [3]. In PD, breakdown of dopaminergic activity at the head of the caudate nucleus is thought to disrupt the functional integrity of the prefrontal cortex and, thus, to be particularly implicated in the occurrence of executive deficits [4,5], although cholinergic [6] and noradrenergic mechanisms [7] are likely to be involved as well. The reduced activation of striato-prefrontal circuits in PD patients has been recently corroborated by functional MRI (fMRI) studies using working memory tasks [8,9]. Memory impairment in non-demented PD has been traditionally viewed as verbal retrieval deficit with verbal recognition being relatively preserved [10,11]. However, recent work indicates that encoding deficits [12-14] and even hippocampal atrophy [15-18] may develop in non-demented PD, independent of verbal retrieval. Hence, while encoding deficits likely mirror mesio-temporal dysfunction, it has been suggested that the verbal retrieval deficit, the hypothetical cause of delayed free recall, is explained by ineffective search strategies normally executed by the intact prefrontal cortex [19], therefore reflecting impaired prefrontal processing. Furthermore and more specifically, strategic or executive deficits are associated with both encoding and retrieval processes, thought to predominantly involve left dorso-lateral prefrontal cortex in the former and right dorsolateral prefrontal cortex in the latter function [20].

As shown in a previous PET study by our group, prefrontal dysfunction seems also to be closely related to impaired somatosensory, i.e. tactile, object discrimination in non-demented PD patients [21]. In this study, patients were engaged in a task of somatosensory discrimination that required repeated encoding, maintenance and retrieval of information for comparison of sequentially explored objects over an extended period of time (see methods section below) and thus demanded a high degree of attention and working memory. In comparison with healthy controls, patients showed poorer somatosensory discrimination performance and reduced implication of the right dorsolateral prefrontal cortex, the dorso-medial thalamus and the mesial frontal cortex, suggesting that frontal association cortices are critically involved in somatosensory information processing. This decline of functional cortico-subcortical connectivity, which was more pronounced in advanced disease stages, might be related to dysfunction of the dorsolateral prefrontal circuit described by Alexander et al. 1990 between thalamus, caudate nucleus and dorsolateral prefrontal cortex, probably due to impaired caudate dopaminergic function [4]. As a probable correlate of impaired prefrontal function, and thus reduced working memory capacity, we observed that our patients had difficulties in dividing attention between following the order of objects explored and discriminating them [5,22]. In a recent fMRI study in healthy normal volunteers we were able to show the prominent role of the prefrontal cortex on working memory during somatosensory discrimination [23].

Here, we present a detailed neurocognitive profile of our PD patient population in relation to prefrontal cortex dysfunction. Our aim was to explore the correlation of impaired capacities for planning, monitoring and retrieving information as assessed by an extensive neuropsychological test battery, with frontal cortex dysfunction as evidenced in the PET study mentioned above and reduced somatosensory discrimination performance. We expected decreased scores in executive functions including deficits in encoding and retrieval processes during delayed free recall in PD patients compared to healthy controls. Furthermore, we hypothesized an interrelation between these tasks and the disrupted prefrontal circuit and parameters of somatosensory discrimination in PD.

## Subjects and methods

### Subjects

Twelve patients with PD (8 males and 4 females, age range from 41 to 66 yrs, mean age 59.1 ± 7.1 (SD)) and 12 healthy subjects (6 males and 6 females, age range from 32 to 64 yrs, mean age 46.6 ± 10.0 (SD)) participated in the study. Informed consent was obtained according to the Declaration of Human Rights, Helsinki, 1975. The study was approved by the Ethics Committee of the Kantonsspital St.Gallen. All subjects had normal MRI brain scans and were right handed as assessed by the Edinburgh Handedness Inventory [24]. PD patients were diagnosed according to UK Brain Bank diagnostic criteria [25]. Disease duration was 7.7 ± 4.1 years (mean ± SD). Disease severity was measured using the Unified Parkinson's Disease Rating Scale (UPDRS), yielding a total score of 36.7 ± 19.3 points (mean ± SD). Psychiatric and medical co-morbidity was excluded in all patients by routine clinical assessment. Treatment in the PD group consisted of a daily levodopa equivalent dose of 766 mg on average. Dopaminergic treatment was optimized and stable at the time of neuropsychological testing and no other centrally active medication was used. Patient characteristics are summarized in Table 1.

**Table 1:** Clinical characteristics

**Disease Duration** ***(yrs, mean ± SD)***	**UPDRS -- Score** ***(points, mean ± SD)***					**SSD Proportion of right answers *(95% C.I.)***
	**Total**	**I**	**II**	**III**	**IV**	
	
7.7 ± 4.1	36.7 ± 19.3	2.6 ± 1.7	15.7 ± 7.6	16.5 ± 6.0	6.3 ± 5.4	0.79 (0.75-0.83)*

UPDRS, Unified Parkinson's Disease Rating Scale (scale maximum 199 points). Subscales in roman numerals: I, Mentation, behavior and mood (subscale maximum 16); II, Activities of daily living (52); III, Motor examination (108); IV, Complications of therapy (23). Higher scores denote increasing disability. SSD, somatosensory discrimination. Comparison with normal volunteers, 0.95 (0.93-0.96, 95% C.I.), unpaired, two-tailed t-test: * p < 0.001. SSD, somatosensory discrimination for object pairs above the critical threshold difference in the major axis of approximately 2 mm

### Neuropsychological evaluation

The neuropsychological evaluation was completed in patients and healthy volunteers before the PET activation study. Prior to the evaluation, PD patients were screened for dementia with the Mini Mental Status Examination (MMSE, cut-off < 27 points) [26].

The same comprehensive neuropsychological test battery was administered to both patients and controls, including the modified Rey Auditory Verbal Learning Test, modified Rey Visual Design Learning Test, Rey Osterrieth Complex Figure Test, phonemic Word Fluency Test, Five Point Test (figural fluency) and Stroop Test [27,28]. The Rey Auditory Verbal Learning Test and Rey Visual Design Learning Test assess immediate memory span, new learning, delayed free recall and recognition for verbal and non-verbal material. We used modified versions with 10 rather than 15 stimulus items, 3 rather than 5 learning trials and a 20-item recognition form. This test battery covers major cognitive domains such as executive functions, as well as figural and verbal memory (see Table 2). According to Lezak et al. [29] we give detailed information about the total score of the learning trials, thought to reflect in part encoding, delayed verbal free recall, thought to reflect retrieval of retained information from episodic memory, and recognition. The Stroop test was scored by measuring the time needed for the color naming trial II subtracted from the color-word interference trial III (Stroop effect) and by counting the errors in trial III.

**Table 2:** Neurocognitive profile of PD patients and normal controls

Test	Patients^#^	Controls	p^§^	Cognitive function
*Executive functions*				

Stroop effect (III-II)	11.4 ± 5.0	10.1 ± 3.4	0.27	Set maintenance, response inhibition, interference
Stroop errors	2.2 ± 1.8	0.7 ± 0.9	0.09	Response inhibition
Verbal fluency	27 ± 8.6	28.3 ± 9.2	0.59	Set maintenance, response inhibition, verbal concept production
Figural fluency	26 ± 9.8	30.6 ± 5.9	0.35	Set maintenance, response inhibition, figural concept production
Rey complex figure copy	17.6 ± 1.0	17.6 ± 1.2	1.00	Visuo-constructive (planning)
Rey complex figure recall	8.1 ± 4.3	11.3 ± 3.4	0.1	Visuo-constructive (planning), figural memory (active)
*Verbal memory				

Verbal learning (mean sum trial I-III)	19.3 ± 3.7	24.1 ± 2.6	**0.002**	Encoding
Immediate memory span (first trial)	5.4 ± 1.0	6.3 ± 1.2	0.1	Working memory
Delayed verbal free recall	4.6 ± 2.9	7.5 ± 1.4	**0.008**	Episodic memory (active)
Verbal recognition	9.2 ± 0.9	9.9 ± 0.3	0.08	Episodic memory (passive)
^+^*Spatial memory*				

Figural learning (mean total)	16.8 ± 5.0	20.1 ± 4.9	0.13	Encoding
Immediate memory span (first trial)	3.8 ± 1.8	5.7 ± 1.7	0.09	Visuo-spatial working memory
Delayed figural free recall	6.2 ± 1.6	7.4 ± 2.0	0.18	Episodic memory (active)
Figure recognition	7.1 ± 2.9	9.0 ± 1.0	0.13	Episodic memory (passive)

^#^Scores are indicated as mean ± SD. ^§^, Mann-Whitney-U, two-tailed. * RAVLT, modified Rey Auditory Verbal Learning Test, ^+^RVDLT, modified Rey Visual Design Learning Test

### Somatosensory discrimination paradigm

The activation test consisted of somatosensory discrimination of three-dimensional shape by the right hand, in which the blindfolded subjects explored and discriminated seven pairs of parallelepipeds that varied only in oblongness, i.e. in the ratio of the major axis to the square base [21]. The objects were made of hard-polished aluminium, had identical volumes and masses (11.5 cm^3^, 32.5 g) and could be easily manipulated with one hand. Parallelepiped pairs were presented such that the differences of the major axes ranged from 0.44 to 5.01 mm, and the differences of the bases from 0.17 to 1 mm. Object presentation followed a two-alternative forced-choice procedure. Each parallelepiped of a pair was presented one at a time to the hand and subjects were instructed to determine with a freely chosen tactile exploration strategy which of the two sequentially presented objects was more oblong. Responses were given non-verbally, i.e. subjects had to extend the thumb of the exploring right hand if the second object of a pair was perceived to be more oblong, or, if this was not the case, to stop exploration and open the hand in order to receive the first object of the next pair. The order of pairs (i.e. length differences) and the order of objects within a pair were pseudorandomized across participants and scanning sessions, implying that length differences were evenly distributed and that the first or second object was longer an equal number of times.

During rest, subjects lay supine in the scanner without cognitive or sensorimotor demand. For analysis of hand movements and responses, each somatosensory discrimination session was recorded on video. From the videotapes, we measured the frequency of thumb movements per second, the total exploration time per object pair and the proportion of correct discriminations as a function of length differences.

### Image data

The PET activation study was performed within one month after completion of the clinical and neuropsychological evaluation.

#### PET image acquisition

On the day of the PET scan, early morning medication was withheld from the patients for an average of 15.7 hours (range: 14 to 18). All subjects were blindfolded and lay supine on the scanner bed. Scanning was performed with a SIEMENS-CTI ECAT 933-04/16 PET-camera (Siemens Knoxville, Tennessee) using oxygen-15-labeled water (H_2_^15^O). Because this camera allows the simultaneous recording of 7 slices only, consecutively acquired cranial and caudal slices were combined to render a data set of 14 axial slices, which covered the brain from the dorsal part of the motor cortex down to the cerebellar nuclei. Planes were reconstructed using a filtered back projection algorithm, with a Hann filter (0.5 Nyquist), which resulted in an 8 mm spatial resolution mm within and between planes. The regional cerebral blood flow was calculated from image and blood data acquired during the first 90 s after arrival of intravenously injected H_2_^15^O in the brain as indicated by a sudden increase in the coincidence counting rate of the PET system [30]; images were acquired for 90 s. Subjects began the task exactly 60 s before injection of the tracer. One data set during the rest condition (rest) and two data sets during somatosensory discrimination were obtained per subject. The two activation scans were averaged before further analysis. While performing somatosensory discrimination the subjects explored several objects implying about nine decisions during image acquisition. The somatosensory discrimination activation lasted around 5 minutes. The order of conditions (somatosensory discrimination, rest) was pseudorandomized. For further details on image acquisition and pre-processing we refer to our previous study [21].

### Statistical analysis

#### Behavioral data

All statistical analyses were performed using SPSS for Windows (Version 15.0.0; SPSS, Inc. Chicago, IL). Neurocognitive performance differences between patients and controls were first evaluated using two-tailed Mann-Whitney-U test for all neuropsychological tests and subtests listed in Table 1. For the somatosensory discrimination task, finger movement frequencies were observed and compared with unpaired, two-tailed t-tests. Furthermore, the probability of correct answers, p[CorrA] for each patient and major axis was estimated to be the ratio of correct answers to the total number of trials at that major axis difference, ΔL. The probabilities for controls and patients were each approximated to a normal distribution for each difference, from which the 95% confidence levels could be determined. Logistic regression (p[CorrA] = 1/1+e^-(d0+d1*ΔL)^) was used to describe the relationship between p[CorrA] and the difference of the cubes. Estimation was carried out using an iterative least squares minimization routine (Eviews (QMS), Systat). Testing statistical hypothesis the constant d_0 _turned out to be zero, and thus, the relationship between the probability of a correct answer and the difference of oblongness of the objects in an individual could be described solely by the coefficient d_1_, a quantitative measure for somatosensory discrimination performance [22].

#### PET data

Preprocessing of the data is described elsewhere [21]. The spatial standardization yielded images consisting of 21 axial image slices 6.43 mm apart, with a matrix of 128 * 128 pixels, each of 2.55 * 2.55 mm. In order to define the neural networks involved in somatosensory discrimination and their configuration in patients and controls, we analysed the regional cerebral blood flow PET data with voxel-based principal component analysis [21]. According to the groups (PD patients, normal volunteers) and conditions (somatosensory discrimination, rest) explored, 48 image volumes were submitted to principal component analysis. Principal component analysis was executed using in-house software written in Matlab [The Mathworks, Inc., Natick, MA] based on the algorithm described in Alexander and Moeller [31].

Principal component analysis in this context is used to extract the covariance structure of the PET image volumes and to identify distributed clusters of voxels that covary in their signal intensity according to experimental condition (somatosensory discrimination, rest) and/or group assignment (patients, controls). Only regional cerebral blood flow values above 30% of the maximal image activity representing the brain are included as pixels in the matrix [32].

Calculation of the residual matrix was the first step. From the matrix of acquisitions are subtracted the subject and group voxel means and added the grand mean, to yield the residual matrix, for which all means vanish. The residual variance was then decomposed into principal components (PCs). Each PC consists of an image, an expression coefficient, and an eigenvalue for each component. The eigenvalue is proportional to the fraction of variance described by each component, the expression coefficients describe the amount that each subject and condition (somatosensory discrimination, rest) contributes to the component, and the component image displays the degree to which the voxels covary in the component. The expression coefficients and voxel values (or voxel loads) of a PC are orthonormal and range between -1 and 1; the orthogonality reflects the statistical independence of the PCs.

Since the expression coefficients can be subjected to statistical tests to indicate the physiological interpretation of the component we used unpaired, two-tailed t-tests on these coefficients to identify PCs that were differentially expressed between healthy controls and PD patients (significance level p < 0.05). The brain areas involved in the group-differentiating PCs were topographically analysed by displaying the PC-load of every voxel with a threshold of 0.5 in a pseudocolor scale on high-resolution MR images. This threshold corresponds approximately to the first and ninety-ninth percentile of voxel values that exhibit the highest correlation with a given PC image. Since the PC load represents the correlation of each voxel with a given component, this procedure allowed the PC pattern to be merged with the anatomy of high-resolution MRI and, thus, to define the centre of gravity of the delineated regions with the highest correlation to a given PC.

#### Correlation of neurobehavioral data with frontal cortex dysfunction and somatosensory discrimination

In the referenced paper [21] we identified three principal components that differentiated PD patients from healthy volunteers (p < 0.05, two-tailed t-test). One of these (PC 7) reflected a distributed cortical-subcortical network during somatosensory discrimination involving prefrontal cortical relay nodes, which indicated its relevance to the present study (Table 3). PC 7 explained 4.7% of the variance in the data and, thus, fulfilled the Kaiser-Guttmann selection criterion [33]. Importantly, according to individual expression coefficients, PC7 was expressed significantly less by PD patients in comparison to normal volunteers during somatosensory discrimination. The individual attributed PC expression scores can be related additionally to subjects' characteristics or external measures of behavior. This procedure allows interpreting group-separating PCs further with respect to individual differences associated with the identified regional patterns [31].

**Table 3:** Group-differentiating principal component 7

PC	Core Anatomical Areas	Talairach Coordinates						PC Load	Group differences*	Functional Correlate
		**R**			**L**					
						
		***x***	***y***	***z***	***x***	***y***	***z***			

PC7	Dorsolateral prefrontal cortex	46	23	22				+	Norm-SSD vs. Patients-SSD (p < 0.02)	Working memory
	Mesial frontal cortex	1	45	23				+		
	Middle temporal gyrus	50	-56	6				+		
	Medio-dorsal Thalamus	2	-14	8				+		
	Medial occipito-temporal gyrus				-5	-80	5	+		
	Insular cortex	49	-9	12				+		
	Superior occipital gyrus	18	-74	6				-		

PC = Principal component. R = right, L = left hemisphere. PC load denotes positive (+) or negative (-) correlation of voxels at the given coordinates with the PC

*Norm-SSD: normal volunteers during somatosensory discrimination (SSD); Patients-SSD: patients during the same condition, unpaired t-test in PC expression coefficient.

Using Spearman's rank correlation test, cognitive function scores that showed significant differences between patients and controls in the Mann-Whitney-U tests of Table 2 (verbal learning and delayed verbal free recall) were correlated with the individual expression scores of PC 7 and the somatosensory discrimination index *d*_1_.

## Results

### Neurocognitive performance

MMSE average score of the patient group was 28.5 ± 1.4 points (mean ± SD). The neuropsychological scores are summarized in Table 2; items are listed according to their corresponding cognitive domain, i.e. executive, verbal and non-verbal memory functions.

The comparison between PD patients and controls revealed significant differences for verbal memory functions. Specifically, PD patients attained significantly lower scores for verbal learning and delayed verbal free recall (both p < .01), whereas immediate memory span and verbal recognition were preserved. In addition, executive functions and figural memory did not show any significant differences.

As the mean ages between PD patients and controls were slightly different, a putative age effect was assessed. However, age had no influence on performances of the verbal learning trials and delayed free recall in both patients and controls. Specifically, regression coefficients for a linear relationship between test performances and age were not different from zero. The hypothesis of a horizontal regression relating these parameters and age could not be rejected, since the observed t-statistics were within the 95%-confidence interval.

### Somatosensory discrimination performance

All participants explored the objects with dynamic digital movements. Consistent with earlier studies, PD patients showed on average a significantly lower thumb movement frequency than the control group (1.4 ± 0.28 versus 2.1 ± 0.4 Hz; unpaired two-tailed t-test, p < .01). Patients also discriminated fewer object pairs per minute (5.4 ± 1.7 versus 6.2 ± 1.7 pairs/min) and required longer exploration times per pair (8.4 ± 3.8 versus 7.6 ± 3.1 sec/pair), but these differences were not significant [21]. This corresponded to 8.1 and 9.2 object pair explorations and decisions in the PD patients and normal volunteers, respectively, during the 90 s acquisition of the somatosensory discrimination task. For object pairs above the critical threshold difference in the major axis of approximately 2 mm, the proportion of correct discriminations was significantly lower in the patient group (0.79, CI 0.75-0.83) compared to the control group (0.95, CI 0.93-0.96) with p < .001 (z-approximation) [21]. On average, the coefficient d_1 _was 0.65 (range 0.34 - 1.06 in the normal volunteers and 0.35 (range 0 - 0.79 in the PD patients (p < 0.01, unpaired, two-tailed t-test). Also for somatosensory discrimination performance, dedicated statistical testing as mentioned above showed no significant age effect in both patients and controls [5]

### Correlation of impaired memory with prefrontal network and somatosensory discrimination

**Figure 1:**
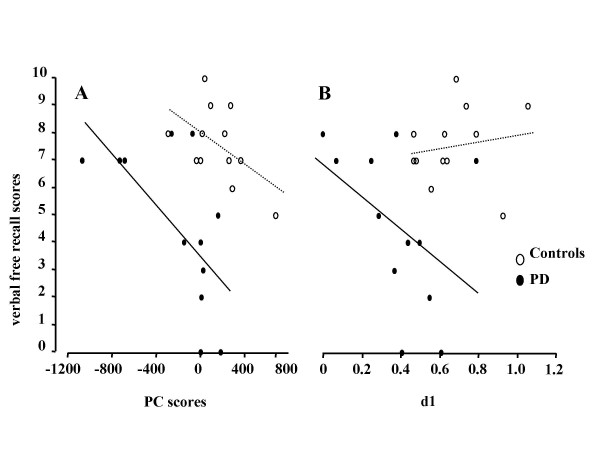
**A. Scatter plot demonstrating significant correlation (p = 0.01) between delayed verbal free recall and PC 7 expression coefficients (=PC scores) in the patient group (filled circles, solid trend line), which were not significantly associated in normal controls (open circles, dotted trend line)**. B. Correlation between delayed verbal free recall and d1 showed a statistical trend (p = 0.08) in patients, but not in normal controls. Note: overall PC7 expression coefficients are on average significantly lower in patients than in normal controls (p < 0.02, two-tailed unpaired t-test).

We found a significant negative correlation between delayed verbal free recall and PC 7 scores in PD patients, but not in the control group (Fig. 1A). It should be emphasized that those individuals who attained a higher score in the delayed verbal free recall score were less likely to engage the neuronal network delineated in PC 7 image during somatosensory discrimination (Table 3). The implication of frontal cortical dysfunction during somatosensory discrimination is indicated by the involvement of the dorsolateral prefrontal cortex and dorsomedial thalamus, i.e. constituents of the dorsolateral prefrontal circuit of Alexander [4], and the mesial frontal cortex. In PD patients a negative correlation at the trend level was also found between delayed verbal free recall and haptic discrimination scores (Fig. 1B). For verbal learning no significant correlations could be found with PC 7 and *d1 *scores, neither in patients nor in controls. Our findings are summarized in Table 4.

**Table 4:** Correlation of impaired verbal memory with prefrontal network (PC7) expression scores and haptic discrimination index d1

		R (Patients, N = 12)	R (Controls, N = 12)
Delayed verbal free recall	PC7	-0.69*	-0.38
	d1	-0.53**	0.31
	
Verbal learning	PC7	0.13	-0.15
	d1	-0.41	0.50

R = Spearman's coefficients of rank correlation, *p = 0.01. **p = 0.08

## Discussion

In the present study, we analyzed the neurocognitive profile of non-demented PD patients with deficient tactile object discrimination and associated abnormalities in a previous PET activation study [21]. We suggested that dysfunctions in prefrontal areas could reflect neurocognitive deficits that ultimately interfere both with neuropsychological test performance and somatosensory information processing. Therefore, executive and memory processes were of main interest in this neurocognitive evaluation, since they support the ability to focus attention and maintain information processing in reaching a decision. Yet, our working hypothesis was that this neuropsychological test battery would supplement our understanding of disrupted executive processes as reported previously in a somatosensory discrimination activation study using PET [21].

Our findings are characterized by a disproportionate impairment of verbal memory during learning and delayed free recall, whereas executive functions and spatial memory are largely preserved. Particularly, set maintenance, held to be impaired frequently in non-demented PD [34-40], was not found to be implicated as indicated by the normal Stroop interference effect and performance in fluency tasks. Intact verbal fluency is noteworthy as it requires efficient retrieval strategies, and also involves the ability to suppress incorrect responses. Nevertheless, the set maintenance ability observed in our PD patients agrees with other studies reporting an intact Stroop interference effect or deficient semantic rather than phonemic fluency [41,42].

Furthermore, sparing of executive functions, notably Stroop interference and verbal fluency, in this small sample suggests that some of our patients might have represented an amnestic type of mild cognitive impairment, as has been recently described [43]. This possibility is also conceivable as our MMSE cut off score (< 27) was not very rigorous. In fact, in a very recent study with a large cohort of non-demented untreated PD patients, the mild cognitive impairment subgroup had average MMSE score of 26.5 [34]. Overall, our findings suggest that neurocognitive functions in mild to moderate stages of PD may be differentially affected and not deteriorate uniformly. The profile of memory deficits is consistent with the traditional view that in non-demented PD, in whom verbal retrieval (reflected by problems in free recall) as a hallmark of fronto-striatal dysfunction is typically more affected than verbal recognition [10,11]. In demented PD patients the pattern may reverse with recognition deficits being more prominent than retrieval impairment [13]. However, recent literature suggests that differences between retrieval and recognition deficits may be less pronounced in PD than originally thought [12,14]. Furthermore, our finding of significantly impaired verbal learning in non-demented PD fits with recent data showing that encoding deficits and hippocampal atrophy may develop early in the disease [12,14-18].

In our earlier studies on the same subjects and at the same time deficient activation of the prefrontal cortex was seen in those patients who were especially deficient in somatosensory discrimination performance and showed low dopaminergic transmission within the caudate nucleus [5,21]. Yet, as a group PD patients showed less engagement of the right dorsolateral prefrontal circuit as evidenced by principal component analysis [21]. The important finding of this study was that somatosensory discrimination deficits correlate strongly with direct evidence of diminished dopamine uptake in the caudate nucleus, and that these deficits are not related to the manual clumsiness normally characteristic of Parkinson's patients [5]. The association of low somatosensory perception and decreased FDOPA-uptake provides direct evidence for the role of the caudate nucleus in the cognitive part of the task, being a relay node of the dorsolateral prefrontal circuit. The findings support the assumption that the PD patients group has a firm neurobiological basis for a neurocognitive failure with disruption of a specific subcortical-cortical circuit involving the prefrontal cortex. The PD patients may thus represent a spectrum of individuals with a relatively preserved to progressively deficient function of the dorsolateral prefrontal cortex during information processing [21]. In a recent study exploring somatosensory discrimination by fMRI we were able to show that the right prefrontal cortex is involved during the phase when retrieval of information from episodic memory is critical for the comparison of sequentially explored objects [44]. For a summary of impaired somatosensory discrimination associated with caudate nucleus dopaminergic transmission (see additional file 1) [5].

PD patients with impaired dorsolateral prefrontal cortex function and associated diminished somatosensory discrimination are impaired also in verbal memory functions. This primary finding of principal component analysis was refined by exploiting within groups the relationship of the PC7, relevant for frontal cortex processing, to the verbal memory functions, the so-called scaled subprofile model to functional imaging [31]. Of note, we found a negative correlation between deficient delayed verbal free recall obtained in the pre-test phase of the PET study and the expression of a somatosensory regional cerebral blood flow covariance pattern showing cortico-subcortical interactions with relay nodes in the mediodorsal thalamus and dorsolateral prefrontal cortex as well as in the mesial frontal cortex. These findings support the concept that verbal retrieval and prefrontal function depend, at least in part, on one another [19]. It should be noted that Fletcher et al. [45] found evidence for the importance of the right dorsolateral prefrontal cortex during retrieval of auditory-verbal material from episodic memory. On an individual basis, the negative correlation shows that the pattern of functional connectivity involving the prefrontal cortex was more expressed in patients with low verbal retrieval scores; conversely, low expression of the prefrontal cortex pattern in the PET study was found surprisingly in patients with better score in verbal retrieval test. This relationship was clearly weaker and insignificant in the control group. Relatively enhanced verbal retrieval might be latent in more advanced PD patients and correspond to silent speaking in order to focus attention during the performance of a specific task. Since it correlates with a network pattern elicited during somatosensory information processing, subvocal verbal cues might have been used also by PD patients during somatosensory discrimination in an attempt to redress impaired prefrontal function. The association of the verbal and haptic domain in PD is further corroborated by the weak correlation at threshold level between delayed verbal free recall and the somatosensory discrimination performance (expressed as *d*_1_). The greater engagement of verbal cues and, hence, of declarative memory processes may be compensatory in nature. Such inverse relationships between neuronal network activities and cognitive processes have been described recently for executive functions in PD patients [46]. In our study, the weak correlation of somatosensory discrimination impairment with deficient verbal free recall also raises the question whether verbal retrieval and tactile object discrimination may be processed by parallel functional neuronal networks converging to common relay nodes. Two regions activated in the network of PC 7, the mesial frontal cortex and the right middle temporal gyrus, might act as these putative points of convergence. Activation of the mesial frontal cortex has been proposed to represent emotional drive related to attention, language and memory; this mechanism could affect both tasks unspecifically [47]. On the other hand, activation of the right middle temporal gyrus might represent a form of subvocal rehearsal during the somatosensory discrimination task.

Interestingly, it has been observed that verbal retrieval strategies of shape information indeed may facilitate tactile object recognition [48]. Moreover, in healthy older adults, the middle temporal gyrus has been shown to increase its activity in the presence of reduced grey matter density in the prefrontal cortex; the increase has been interpreted as a compensatory mechanism for prefrontal dysfunction [49]. Similarly, a shift to the declarative memory system in PD during planning tasks, possibly resulting from insufficient working memory capacity within the fronto-striatal system, has been reported [8], i.e. PD patients activated alternatively the hippocampus which also projects to the prefrontal cortex. Summarizing, we propose that compensatory mechanisms might be of significance in the presence of prefrontal dysfunction through (1) unspecific enhancement via the mesial frontal cortex and (2) verbal rehearsal strategies via the middle temporal gyrus.

There are limitations in our study. Firstly, the sample size was small increasing the risk of type II errors, and many statistical tests were conducted increasing the chance of type I error. After Bonferroni correction of the alpha-level for multiple comparisons the verbal free recall deficit in PD patients would just fail to reach the significance level when compared with healthy controls. On the other hand, we think that the significant correlation of verbal free recall with the PET data in PD patients underscores the reliability of the finding rendering the strict correction an inacceptable risk of type II error. Secondly, our findings may be influenced by the fact that the patients were not in defined OFF state during neuropsychological testing in contrast to the PET scanning. However, we would expect that the correlation of the PET data with the neurocognitive variables might have been stronger if the experimental conditions were controlled more strictly. Thus, our observations support the assumption that the negative correlation between verbal retrieval and the dorsolateral prefrontal circuit reflects compensation for impaired routines in PD patients.

## Competing interests

The authors declare that they have no competing interests.

## Authors' contributions

SB participated in the design of the study, performed the statistical analysis and wrote the manuscript. EA participated in data analysis and reviewed the manuscript. DW evaluated the neuropsychological testing. BW designed the study, reviewed the statistical analysis and wrote the manuscript. All authors read and approved the final manuscript.

## Supplementary Material

Additional file 1 **Somatosensory discrimination and caudate nucleus metabolism**. Additional file 1 summarizes a previous study on the correlation between impaired Somatosensory discrimination and reduced FDOPA-uptake in the caudate nucleus. Click here for file
