# Supplementary material for: Impaired verbal memory in Parkinson disease: relationship to prefrontal dysfunction and somatosensory discrimination
Source: Behav Brain Funct. 2009 Dec 15;5:49. doi: 10.1186/1744-9081-5-49 (PMC2805678; doi:10.1186/1744-9081-5-49)
Supplement: Additional file 1 — Somatosensory discrimination and caudate nucleus metabolism. Additional file 1 summarizes a previous study on the correlation between impaired Somatosensory discrimination and reduced FDOPA-uptake in the caudate nucleus. [file 1744-9081-5-49-S1.DOC]

Additional File 1: Somatosensory discrimination and caudate nucleus metabolism

In the same PD patients group as described in the body of the manuscript, a FDOPA-PET study was performed within one month after the PET-somatosensory discrimination study. For detailed description of method, data analysis and results we refer to ref. 5 in the main text.

The uptake-rate constant (Kc) was calculated in defined target regions (e.g. putamen, caudate nucleus and medial prefrontal cortex) and reference regions (occipital cortex). Kc was calculated for each target region of interest (ROI) from the slope derived from a graphical analysis method [a1], using the occipital lobe ROI as brain reference. The findings were compared to the reference data of our laboratory consisting of 12 normal volunteers ranging in age from 50 to 60 years [a2].

Kc was significantly diminished for the putamen in all PD patients and for the caudate nucleus in seven PD patients (subgroup B in Table A1 below). A detailed analysis indicated an essential influence of the FDOPA uptake in the caudate nucleus upon subgroup categorization whereas the influence of the FDOPA uptake in the putamen was insignificant. This is in accordance with similar motor parameters observed in both subgroups, i.e. subjects with normal and decreased FDOPA uptake in the caudate nucleus (see ref. 20, main text). Uptake-rate constants of FDOPA for the putamen and caudate nucleus are integrated in Table 5, containing clinical and somatosensory discrimination data, and detailed according to the subgroups with normal and decreased uptake in the caudate nucleus.

| Table A1 |  |  | | | | |  | |  |
| --- | --- | --- | --- | --- | --- | --- | --- | --- | --- |
| Group | **Duration**  **of disease^1^**  **(mean ± SD yrs)** | **UPDRS - Score^2^** | | | | | **FDOPA-uptake (Kc)^3^** | | **SSD^4^** |
|  |  | **Total** | **I** | **II** | **III** | **IV** | **Putamen** | **Nc.caud.** | **Right answer**  **Proportion (95% C.I.)** |
|  |  | **(mean ± SD)** | | | | | **(mean ± SD)** | |  |
| **All patients**  **(n=12)** | 7.7 ±4.1 | 36.7 ± 19.3 | 2.6 ± 1.7 | 15.7 ± 7.6 | 16.5 ± 6.0 | 6.3 ± 5.4 | 0.0044 ± 0.0019 | 0.0073 ± 0.0018 | 0.79(0.75-0.83) $ |
| **Subgroup A**  **(N = 5)** | 4.6±2.2 | 19.0 ± 10.5 | 1.6 ± 1.7 | 9.8 ± 3.8 | 15.6 ± 4.7 | 2.6 ± 5.0 | 0.0054 ± 0.0024 | 0.0091 ± 0.0007 | 0.85(0.78-0.90) $ |
| **Subgroup B**  **(N = 7)** | 9.9*±4.4 | 49.3* ± 12.7 | 3.3 ± 1.4 | 19.9* ± 6.9 | 17.1 ± 7.1 | 19.0* ± 4.9 | 0.0037 ± 0.0011 | 0.0064* ± 0.001 | 0.75(0.70-0.81) */$ |
| **Controls**  **(N = 12)** |  |  |  |  |  |  | 0.0097 ± 0.0008 | 0.0114 ± 0.0012 |  |

Comparison of subgroups A an B, * indicates significant differences in the unpaired, two-tailed t-test :

^1^ Duration of disease: p<0.05

^2^ Unified Parkinson’s disease rating scale, total: p<0.01; 4 components: I.) Mentation, behavior and mood; II.) Activities of daily living: p<0.05; III.) Motor examination; IV.) Complications of therapy: p<0.05

^3^ FDOPA-uptake in the caudate nucleus: p<0.001

^4^ Somatosensory discrimination (SSD) performance for object pairs above the critical threshold difference in the major axis of approximately 2 mm: p<0.05

Significant impairment in the comparison of normal volunteers 0.95(0.93-0.96) at $ p < 0.001 (z-approximation)

References

a1. Antonini A, Vontobel P, Psylla M, Günther I, Maguire RP, Missimer J, Leenders KL. Complementary positron emission tomographic studies of the striatal dopaminergic system in Parkinson’s disease. Arch Neurol 1995, 52: 1183-1190.

a2. Patlak CS and Blasberg RG. Graphical evaluation of blood-to-brain transfer constants from multiple-time uptake data: generalizations. J Cereb Blood Flow Metab 1985, 5: 584-590.
